# Physical Degeneration

**Published:** 1905-09-09

**Authors:** 


### Editor's Letter-Box.

[Our Correspondents are reminded that prolixity is a great bar to blication, and that brevity of style and conciseness of statement greatly facilitate early insertion.]

### PHYSICAL DEGENERATION.

"C. F., M.R.C.S. and L.R.C.P.," writes as follows: As a medical man of 30 years' experience let me protest against the term "physical deterioration." It is absolutely uncalled for as regards Londoners. Having practised for many years in the North of England I found consumption and lunacy common in all villages. Since my return to London I have practised in the East End, Ilford and Bromley, and can honestly say that I never saw a finer growing generation. The physique of the men is remarkable for size, height, and spirits. I have examined the children, and nothing more can be desired. They are generally full of health, and well set up. Country children could not compare with them (plentiful and cheap food accounts for this). Were it not for the infantile mortality London would be the healthiest city in the world.

Now let us take the causes. First I should say it must be attributed to drunken women—and they are terribly on the increase. Then charitable institutions, hospitals and nursing institutions. These women drag their children backwards and forwards to the hospitals, shifting their responsibilities which they can well afford to pay. These competing charities in the last stage of illness take them in, and so these mothers escape a coroner's inquest, and with their husbands' wages buy more drink. These are the two factors which have to be met, nothing else.

